# Severe clinical outcome is uncommon in *Clostridium difficile* infection in children: a retrospective cohort study

**DOI:** 10.1186/1471-2431-14-28

**Published:** 2014-01-31

**Authors:** Kevin L Schwartz, Ilyse Darwish, Susan E Richardson, Michael R Mulvey, Nisha Thampi

**Affiliations:** 1 Division of Infectious Diseases, The Hospital for Sick Children, University of Toronto, 555 University Ave, Rm 7306, Toronto, ON M5G 1X8, Canada; 2 Sandra A. Rotman Laboratories, University Health Network, University of Toronto, Toronto, ON, Canada; 3 Division of Microbiology, Department of Paediatric Laboratory Medicine, The Hospital for Sick Children, University of Toronto, Toronto, ON, Canada; 4 National Microbiology Laboratory, Winnipeg, MB, Canada; 5 Division of Infectious Diseases, Mount Sinai Hospital, University of Toronto, Toronto, Canada

**Keywords:** *Clostridium difficile*, North American pulsed-field type, Pediatrics, Child

## Abstract

**Background:**

*Clostridium difficile* infection (CDI) is the most common cause of health care–associated diarrhea in children and adults. Although serious complications of CDI have been reported to be increasing in adults, this trend has not yet been demonstrated in children. The purpose of this study was to examine the features of CDI in a pediatric population, with special attention to the occurrence of CDI-related severe outcomes.

**Methods:**

A chart review was conducted for patients with *C. difficile* infection detected by cytotoxin assay between August, 2008 and July, 2012. Basic demographics, mode of acquisition (nosocomial versus community), laboratory and clinical features, treatment, and outcome data were collected. Pulsed-field gel electrophoresis and polymerase chain reaction detection of toxin A (*tcdA*), toxin B (*tcdB*), binary toxin (*cdtB*) and *tcdC* genes were performed on isolates from nosocomial cases by the National Microbiology Laboratory, Winnipeg, Manitoba.

**Results:**

Ninety percent of children with CDI experienced resolution of symptoms by 30 days after disease onset and 2% experienced a severe outcome. There were no cases where colectomy was performed for CDI, and only one case where CDI contributed to death. Various combinations of clinical and laboratory features were not predictive of a severe outcome. Seventy-four percent of cases were nosocomial-associated. Among all cultured strains, the NAP4 strain occurred most frequently (24%), followed by NAP1 (11%). There was no association between strain type and clinical outcome; however, relapses were significantly more frequent in NAP4-infected children.

**Conclusions:**

Severe outcomes due to CDI are uncommon in children compared to adults. Further prospective pediatric studies on CDI in community and hospital settings are required to better understand risk factors, optimal treatment and the significance of NAP4 in pediatric CDI.

## Background

*Clostridium difficile* is the most common cause of diarrhea in hospitalized children [1,2], usually associated with antibiotic use. Over the last decade incidence rates of nosocomial *C. difficile* infection (CDI) in adults have increased in most geographical locations [3]. Accompanying the rise in incidence has been an increase in the rate of colectomies performed for CDI, length of stay (LOS), hospital costs, and attributable mortality [4-6] associated with *C. difficile* infection. This rise in severity has been largely linked to the emergence of the North American pulse-field gel electrophoresis type 1, restriction endonuclease analysis group BI, polymerase chain reaction (PCR) ribotype 027 (NAP1/BI/027) strain [5,7].

The epidemiology of pediatric *C. difficile* has been less well characterized, however recent data suggest a rise in the incidence rate of nosocomial CDI, from 4.4 to 6.5 cases per 10,000 patient-days in the United States (US) between 2001 and 2006 [8]. Other authors have documented a rise in incidence of 9% [9] to 14% [10] per year. A similar rise in community-associated cases has also been observed [11,12]. One U.S. pediatric study showed an increase from 2.2 to 23.5 cases of CDI per 100,000 persons over the previous two decades [12]. Despite the more frequent diagnosis of CDI, there has not been an increase in disease severity as measured by LOS, colectomy rate, and mortality rate [9,10,13]. However, longer LOS and higher colectomy rates occur in children with CDI compared to non-CDI hospitalized controls matched for demographics and comorbid conditions [9].

Given our clinical observation that CDI in children is rarely associated with a severe clinical outcome, as compared to adults, we set out to characterize severe outcomes and CDI-associated complications at a pediatric hospital providing primary (self-referred cases) to tertiary (regional referral center) care to a large population base.

## Methods

A retrospective chart review was conducted at a 370-bed pediatric academic hospital in Toronto, Canada. Our institution services a varied pediatric population, ranging from the well child to the immune compromised child. This hospital performs 110 autologous and allogeneic hematopoietic stem cell transplants annually, in addition to solid organ transplants (liver, kidney, heart, lung, and small bowel). Patients from 1 through 17 years of age with a positive *C. difficile* cytotoxin assay in the stool between August 1, 2008 and July 31, 2012, were identified from microbiology records. Our laboratory restricts testing to children greater than one year of age.

Charts from the first presentation of CDI were reviewed and information related to basic demographics, laboratory and clinical features, *C. difficile*-directed antibiotic use, and clinical outcome were extracted. Clinical and laboratory features included stool frequency and consistency. Clinically-based definitions for diarrhea were modified from the Canadian Nosocomial Infection Surveillance Program (CNISP) definitions [14] for use in children, and were defined as: severe: 6 or more watery stools in a 36-hour period; moderate: greater than 2 loose stools, but not meeting the definition for severe; mild: a change in stooling pattern to 1–2 loose stools daily; no diarrhea or not documented. Risk factors for severity of infection were defined as: presence of blood in stool, fever ≥38°C, leukocytosis ≥15×10^9^/L, hypoalbuminemia ≤25 g/L, and creatinine above the normal limit according to age [15]. A severe outcome was defined as the need for intensive care unit (ICU) admission, colectomy, or death within 30 days of a positive *C. difficile* cytotoxin assay [14]. Each severe outcome was evaluated and designated as unrelated to, contributing to, or a direct effect of CDI by authors, KS and ID, and confirmed by two other investigators, SR and NT. Other clinical outcomes assessed at 30 days after disease onset included date of discharge from hospital, presence or absence of symptoms, or ongoing treatment directed at *C. difficile*. Relapse was defined as recurrence of previously resolved symptoms with a positive test for *C. difficile,* two or more weeks from the onset of the initial episode, whether or not the patient had been treated with antibiotics. If patients required re-admission for relapse, their charts were reviewed to capture any severe outcome, however, each patient was only included in the study once. *C. difficile* was categorized as community-associated if symptoms developed within 72 hours of admission, with no hospitalization in the previous 8 weeks, consistent with CNISP [14]. This study was approved by the Quality Improvement Review Board at the Hospital for Sick Children, Toronto, Canada. Individual patient/guardian consent was not obtained.

Statistically significant differences between groups were assessed by the chi-squared test or Fisher’s exact test, where appropriate. A p-value <0.05 was considered statistically significant. The incidence of nosocomial-associated disease was calculated to be the number of cases of CDI per 10,000 hospital inpatient days.

### Laboratory methods

*C. difficile* toxin was detected using a Vero cell cytotoxicity assay as previously described [16]. Stools from nosocomial CDI cases were frozen at -80°C, and referred to the National Microbiology Laboratory, Winnipeg, Canada, for culture, typing and further analysis as part of a larger study through CNISP [14]. Isolates were typed using pulsed-field gel electrophoresis (PFGE) to determine North American pulsed-field (NAP) types, and PCR to detect the presence of genes for toxin A and B (*tcdA* and *tcdB*, respectively), binary toxin (*cdtB*), and size alterations of the toxin gene regulator (*tcdC*) [7]. Minimum inhibitory concentrations of each strain to a panel of antibiotics (metronidazole, clindamycin, vancomycin, rifampin, moxifloxacin, tigecycline) were determined by the Etest method [17].

## Results

From August 1, 2008 to July 31, 2012 there were 5751 stool samples tested for *C. difficile* toxin by cytotoxin assay, with 435 positives and 299 individual patients analyzed. During the study period, the nosocomial CDI rate was 8.3 per 10,000 patient days. This trend did not change in any age group or year over the 4-year study period. Hospital-associated infections accounted for 222 (74%) of cases, and 77 (26%) were community-associated.

Demographic, clinical and laboratory findings are summarized in Table 1. Males accounted for 58% of the total number of cases and the median age was 6.5 years (interquartile range, 3–13 years). Nearly 40% of cases had an underlying malignancy, a third of which had undergone hematopoietic stem cell transplant (HSCT). Inflammatory bowel disease (IBD) comprised 10% of cases. Among children with IBD, 55% (17/31) had community-associated disease.

**Table 1:** **Characteristics of 299 children with *Clostridium difficile *** **cytotoxin detected in stool**

**Category**	**n (%)**
**Demographic Characteristics**	
Age (years)	6.5 (3–13)^†^
1–5	125 (42)
6–11	83 (28)
12–17	91 (30)
Male	173 (58)
**Underlying medical condition**	206 (69)
Malignancy	78 (26)
Hematopoietic stem cell transplant	40 (13)
Chromosomal/genetic syndrome	33 (11)
Inflammatory bowel disease	31 (10)
Solid organ transplant	24 (8)
Cystic fibrosis	8 (3)
Other*	8 (3)
Congenital heart disease	6 (2)
Hirschsprung’s disease	2 (1)
**Hospital-associated CDI**	222 (74)
**Clinical features**	
Fever ≥ 38.0°C	140 (47)
Abdominal pain	106 (35)
Bloody stool	51 (17)
Ileus	7 (2)
Diarrhea	
Severe	81 (27)
Moderate	132 (44)
Mild	65 (22)
None/no documentation	21 (7)
**Laboratory features**	
White blood cells ≥ 15 × 10^9^/L	64 (21)
Creatinine > normal level (adjusted for age)	40 (13)
Albumin ≤ 25 g/L	45 (15)
**CDI-directed antimicrobial therapy**	240 (80)

^†^Median (interquartile range).

*Other underlying medical conditions: Aplastic anemia (4), Juvenile idiopathic arthritis (3), and biliary atresia (1).

Mild, moderate, or severe diarrhea was seen in 22%, 44%, and 27% of children with CDI, respectively (Table 1). Clinical severity was similar in community- and nosocomial-associated cases. Two or more risk factors for severe disease were noted among 89 children (30%). However, these cases were not significantly associated with severe outcomes, compared to those who had less than two risk factors (Table 2). The relapse rate was 17% overall. Eighty per cent of children with CD toxin in their stool received antibiotic therapy directed at CDI, usually metronidazole.

**Table 2:** **Outcomes of 299 children with *C. difficile *** **infection**

**Outcome**	**Total, n (%)**	**2 or more risk factors**^**†**^**, n (%)**	**CDI treatment received, n (%)**	**Underlying diagnosis of IBD, n (%)**	**Underlying diagnosis of cancer/following BMT, n (%)**
**Discharged or resolution of symptoms**	269 (90)	77 (29)	211 (71)	22 (8)	107 (40)
**Ongoing diarrhea and/or treatment**	28 (9)	12 (43)	27 (96)	9 (32)*	7 (25)
**ICU admission**	5 (2)	3 (60)	5 (100)	0 (0)	4 (80)
**Death**^**‡**^	1 (0.3)	1 (100)	1 (100)	0 (0)	1 (100)
**Relapse**	52 (17)	13 (25)	43 (83)	5 (10)	24 (21)

*Significant at p < 0.05. Children with IBD and CDI were more likely to have ongoing diarrhea compared to children without IBD by Chi-squared test. No other significant differences were identified.

^†^Risk factors include fever >38°C, bloody stool, hypoalbuminemia ≤25 g/L, leukocytosis ≥15×10^9^/L, and renal dysfunction defined as creatinine above normal limits for age. There were no significant differences in outcomes (p > 0.05) in children with and without 2 or more risk factors.

^‡^Death to which CDI contributed.

CDI = *C. difficile* infection; ICU = intensive care unit; IBD = inflammatory bowel disease; BMT = bone marrow transplant.

At thirty days following the onset of symptoms, 89% of patients were discharged or had resolution of symptoms, 2% had a severe outcome (5 ICU admissions, 1 CDI-associated death), and 9% had ongoing diarrhea or were still receiving treatment, more than half of whom had underlying IBD or malignancy (Table 2). The 5 children admitted to the ICU required management of ileus, upper gastrointestinal bleeding, or septic shock. Colectomy was not performed for any reason on any patient in the cohort during the study period.

There was one death during the study period, to which CDI was deemed to contribute. This was in a 7-year-old child following HSCT who developed severe diarrhea followed by septic shock and ileus. He was admitted to the ICU, grew *Enterobacter cloacae* from his blood with *C. difficile* toxin identified in the stool on the same day. The child was unstable and no surgical intervention was considered. Within 48 hours of admission to ICU the child died from severe sepsis. The primary cause of death was felt to be gram-negative sepsis with CDI contributing to death.

PFGE data were available for 90 cultured *C. difficile* isolates from 129 nosocomial cases submitted to CNISP between August 1, 2008 and July 31, 2012 (Table 3). Thirty-four isolates (38%) did not have a fingerprint pattern that fell into a NAP cluster designation. NAP4 was the most common strain type (n = 22, 24%), followed by NAP1 (n = 10, 11%). Relapse rates were 41% and 15% for NAP4 and non-NAP4 strains, respectively, which was statistically significant (p = 0.009). There were no significant differences in symptom severity, the need for ICU admission, surgical consultation, or death based on strain type. Of the 90 isolates, all had toxin A and B detected by PCR. Binary toxin was detected in 16 (18%) of 90 isolates, and in 9 out of 10 of the NAP1 strains. The *tcdC* gene was detected in all isolates; all NAP1 strains had the characteristic 18 bp deletion. All isolates were susceptible to metronidazole, vancomycin, rifampin, and tigecycline. In addition, 60% of isolates were intermediate or resistant to clindamycin, and 9% were intermediate or resistant to moxifloxacin. Of the NAP1 and NAP4 strains, 30% and 9% were intermediate or resistant to moxifloxacin, respectively (Table 3).

**Table 3:** **North American Pulsed-field types in 90 children with nosocomial *C. difficile *** **infection and organism available for testing**

**Strain**	**Total, n (%)**	**Relapse, n (%)**	**Severe outcome**^**†**^**, n (%)**	**Moxifloxacin susceptible, n (%)**
**NAP4**	22 (24)	9 (41)*	0 (0)	20 (91)
**NAP1**	10 (11)	1 (10)	1 (10)	7 (70)
**Other NAP type**^**‡**^	24 (27)	2 (8)	0 (0)	22 (92)
**No NAP type**	34 (38)	7 (21)	2 (6)	33 (97)
**Total**	**90**	**19 (21)**	**3 (3)**	**82 (91)**

*p = 0.009.

^†^Severe outcome = ICU transfer, colectomy, or death within 30 days of a positive *C. difficile* cytotoxin assay.

^‡^Other NAP = NAP10 (n = 4), NAP11 (n = 8), NAP6 (n = 3), NAP7 (n = 2), NAP2 (n = 3), NAP8 (n = 2), NAP12 (n = 2).

## Discussion

In this study, we describe a cohort of 299 hospitalized children, 1–17 years of age, with *C. difficile* infection at a large tertiary care centre. Three quarters of the cases were nosocomial in origin and occurred among children with chronic underlying medical conditions, many of whom had compromised immune systems or inflammatory bowel disease. We found that severe clinical outcomes related to CDI occurred in only 2% of our cases, despite the presence of serious underlying illness in 50% of cases. Colectomy was not performed in any child, few were admitted to the ICU, and CDI was felt to contribute to only one death in the cohort. Most children fully recovered from their illness by 30 days after disease onset, irrespective of whether the illness was nosocomial or community-associated, the severity of illness, or treatment received. Of the 9% with ongoing diarrhea, more than half had underlying IBD or malignancy, which may have confounded the determination of etiology of persistent symptoms. We also observed a high prevalence of NAP4 among typeable isolates in our pediatric cohort, which is in contrast to the high prevalence of NAP1 strains in adults from similar geographic areas [18].

The incidence rate of nosocomial CDI (8.3 cases per 10,000 patient days) did not change significantly over the study period. This is in contrast to studies of American children, in which a 9% yearly rise in incidence of CDI has been reported [8,19].

The severity and outcome of CDI in children is poorly understood compared to adults. Pediatric data comparing children with CDI to matched controls have shown longer LOS, higher hospital costs, and higher rates of colectomy [8,9]. Administrative data sets including thousands of children with CDI have indicated colectomy rates ranging from 0.35 to 1.2% [8,9,20]. Adjusted for comorbidities, children with CDI in a large database study had a 1.36 fold increased risk of undergoing colectomy compared to controls without CDI [9]. One of the challenges with such administrative database studies is ascertaining the reasons for colectomy. It is possible that a number of the colectomies were performed for underlying IBD or for another clinical indication. If *C. difficile* is detected during an IBD exacerbation, it has been shown to be a marker for severity and increased in-hospital mortality [21]. However, among both pediatric patients with IBD and non-IBD controls, toxigenic *C. difficile* is frequently detected irrespective of symptoms [22]. As a result, some of the complications seen in pediatric CDI are possibly accounted for by underlying comorbidities.

In our study, a small number of children (2%) suffered a severe outcome. This is in marked contrast to adult CDI, where severe outcomes have been reported in 8–20% [7,23] and *C. difficile*-attributed or associated mortality has been reported in approximately 5–9% of cases, especially among those harboring the NAP1 strain [4,7,24,25].

In contrast to reports of adult CDI, NAP4 was the most common strain type in our pediatric cohort, accounting for 24% of nosocomial isolates. NAP1 was the second most common at 11%. Similar rates of NAP1 have recently been reported among children in the US [26,27]. The trend across Canada in recent years has been toward an increasing incidence of NAP1 among adults with CDI, from 28.5% (2005) to 43.5% (2010), with the highest prevalence of 51% in eastern Canada [18].

Neither NAP4 nor NAP1 was associated with more severe illness in our cohort, although relapse rate was significantly higher in patients with NAP4 compared to non-NAP4 strains. However, we could not determine whether the relapses were caused by the same strain. In contrast, a large Canadian study of adult CDI showed that severe outcomes occurred significantly more frequently in patients between the ages of 60 and 90 years of age who were infected with the NAP1 strain, compared to other strains [7]. However, the importance of the NAP1 strain in severity of disease has been challenged by case–control studies that were unable to demonstrate any difference in disease severity with NAP1 infection compared to other strains [23]. From our data we were unable to associate the high prevalence of NAP4 with a cluster of cases, limited in time or geography. As this is the first report of NAP4 predominance in pediatric CDI, further investigation will be necessary to elucidate the role of NAP4 in disease in children and to better determine associations with disease relapse.

Another important difference between children and adults in Canada is the rate of moxifloxacin susceptibility. In our cohort 91% of all strains (91% of NAP4 and 70% of NAP1 strains) were susceptible to moxifloxacin. In contrast, only 33% and 17% of all strains, and NAP1 strains, respectively, were susceptible to moxifloxacin in a predominantly adult Canadian study [14]. It should also be noted that the PFGE subtype of the NAP1 isolates identified in this study were not the common NAP1 PFGE subtype observed across Canada which is typically moxifloxacin-resistant. The lower use of fluoroquinolones in pediatrics could be driving this observation.

There are no established pediatric predictors for disease severity. Our study demonstrated that proven adult risk factors of fever, bloody stools, leukocytosis, renal dysfunction, and hypoalbuminemia were common in our population. These so-called risk factors were not associated with disease severity or complications and were likely related to the underlying conditions of malignancy and IBD. This finding has been identified by other authors who have proposed new criteria for pediatric CDI, which require further study and validation [28].

Most infections were associated with current or previous hospitalization, and occurred predominantly among children with underlying immunocompromising conditions. Community-associated infection was observed in 26% of children, more than half of whom had IBD. These findings represent our population of children with high rates of comorbid conditions. Community-associated disease is harder to assess in this setting and would require a community-based study to determine incidence. A recent population-based study in the U.S. identified that 75% of pediatric CDI was community-associated, suggesting that hospital-based incidence rates likely underestimate true CDI rates in children [12]. However, in our study, we were unlikely to miss complicated cases, as we expect most of these children to have been referred to our centre from the community. Given the lack of data and the increasing incidence of community-associated disease [11,12], future pediatric CDI research should focus on this issue.

Our study has several limitations and biases inherent to retrospective reviews. Findings were limited to information available from the charts. Almost 30% of children in this study would not have met adult criteria for disease by having less than 3 loose stools per day. However as there are no well-defined pediatric criteria for significant disease and, as the goal of this study was to detect any CDI related complication, we included all children with *C. difficile* detected in stools sent for clinical reasons. The cytotoxic assay is very specific and correlates well with disease but lacks sensitivity compared to PCR or to cytotoxic culture (toxin assay on culture positive *C. difficile*). Therefore it is possible some cases were missed. Sixty percent of nosocomial isolates were sent for reference typing, but in only half of these *C. difficile* was recovered on culture, resulting in a complete clinical with strain-typing dataset of 30% of the included children in the study. Antibiotic exposure data was missing for too many patients from which to draw meaningful conclusions regarding specific risk factors, particularly in community-associated disease. This will be important data to collect in future prospective studies on CDI in children.

Our study has a number of strengths. We were able to show the relationship of pediatric CDI with generally benign clinical outcomes. Furthermore since our hospital excludes testing in infants less than 1 year of age and our laboratory was utilizing a cytotoxin assay during the study period, we have confidence that these cases had a high probability of representing true infection as opposed to colonization.

## Conclusions

This study describes a population of 299 children with nosocomial and community-associated CDI over a four-year period. Children had a much lower rate of severe complications of CDI than that reported in adults. This is the first report to describe NAP4 as the predominant nosocomial strain type in a pediatric institution, and its association with relapse.

## Competing interest

The authors declare that they have no competing interests.

## Authors’ contributions

KS drafted the manuscript, assisted in collecting and analyzing data. ID collected and analyzed data as well as assisted in editing. SR assisted in design of the study, coordination, and helped draft and review the manuscript. MM performed the sequencing, PCR, and sensitivity analysis. NT assisted in design of the study, data collection, and helped draft and review the manuscript. All authors contributed significantly to editing the manuscript and approved it in its current form.

## Pre-publication history

The pre-publication history for this paper can be accessed here:

http://www.biomedcentral.com/1471-2431/14/28/prepub
